# Discrimination of ivory from extant and extinct elephant species using Raman spectroscopy: A potential non-destructive technique for combating illegal wildlife trade

**DOI:** 10.1371/journal.pone.0299689

**Published:** 2024-04-24

**Authors:** Rebecca F. Shepherd, Adrian M. Lister, Alice M. Roberts, Adam M. Taylor, Jemma G. Kerns

**Affiliations:** 1 Bristol School of Anatomy, University of Bristol, Bristol, United Kingdom; 2 Faculty of Health and Medicine, Lancaster University, Lancaster, United Kingdom; 3 Natural History Museum, London, United Kingdom; 4 University of Birmingham, Edgbaston, Birmingham, United Kingdom; University of Gothenburg: Goteborgs Universitet, SWEDEN

## Abstract

The use of elephant ivory as a commodity is a factor in declining elephant populations. Despite recent worldwide elephant ivory trade bans, mammoth ivory trade remains unregulated. This complicates law enforcement efforts, as distinguishing between ivory from extant and extinct species requires costly, destructive and time consuming methods. Elephant and mammoth ivory mainly consists of dentine, a mineralized connective tissue that contains an organic collagenous component and an inorganic component of calcium phosphate minerals, similar in structure to hydroxyapatite crystals. Raman spectroscopy is a non-invasive laser-based technique that has previously been used for the study of bone and mineral chemistry. Ivory and bone have similar biochemical properties, making Raman spectroscopy a promising method for species identification based on ivory. This study aimed to test the hypothesis that it is possible to identify differences in the chemistry of mammoth and elephant ivory using Raman spectroscopy. Mammoth and elephant tusks were obtained from the Natural History Museum in London, UK. Included in this study were eight samples of ivory from *Mammuthus primigenius*, two samples of carved ivory bangles from Africa (*Loxodonta species*), and one cross section of a tusk from *Elephas maximus*. The ivory was scanned using an inVia Raman micro spectrometer equipped with a x50 objective lens and a 785nm laser. Spectra were acquired using line maps and individual spectral points were acquired randomly or at points of interest on all samples. The data was then analysed using principal component analysis (PCA) with use of an in-house MATLAB script. Univariate analysis of peak intensity ratios of phosphate to amide I and III peaks, and carbonate to phosphate peaks showed statistical differences (p<0.0001) in the average peak intensity ratios between *Mammuthus primigenius*, *Loxodonta spp*. and *Elephas maximus*. Full width at half maximum hight (FWHM)analysis of the phosphate peak demonstrated higher crystal maturity of *Mammuthus primigenius* compared to living elephant species. The results of the study have established that spectra acquired by Raman spectroscopy can be separated into distinct classes through PCA. In conclusion, this study has shown that well-preserved mammoth and elephant ivory has the potential to be characterized using Raman spectroscopy, providing a promising method for species identification. The results of this study will be valuable in developing quick and non-destructive methods for the identification of ivory, which will have direct applications in archaeology and the regulation of international trade.

## Introduction

The trade in elephant ivory is a global issue contributing to the decline of elephant populations worldwide. While many countries have in recent years restricted their laws on ivory trade, and most have banned all trade, there are often exceptions for antique items or items of cultural significance [1]. However, these ivory trade rules do not apply to extinct species such as mammoth ivory [1]. The trade of mammoth ivory is on the increase with the rise of ‘mammoth hunters’ undertaking expeditions through the Siberian arctic to harvest mammoth tusks for financial gain [2]. This activity has been made easier during recent years, as an increase in global temperatures has resulted in thawing of the permafrost [3], revealing almost perfectly preserved mammoth specimens during the summer months. This legal source of ivory poses an enforcement problem for border protection and customs teams across the globe, as ivory products of these two different types of tusk can be difficult to distinguish from one another visually [4].

Tusks are a mineralised connective tissue [5] formed from layers of cementum, enamel and dentine, with a medullary pulp cavity occupying the proximal tusk [6]. Dentine comprises the bulk of tissue in the tusk. Its microarchitecture involves dentinal tubules radiating from the pulp cavity to the cementum [7, 8]. Dentine is formed by odontoblasts, which move centrally as they lay down matrix, and line the medullary cavity; these cells produce collagens type I and IV [9] which are subsequently mineralised by calcium phosphate based dentinal apatite crystals [10]. In a section of tusk, a checkerboard pattern of dark and light lines may be seen radiating from the centre to the periphery. This ‘Schreger pattern’ is thought to relate to minute shifts in the path of odontoblasts as they deposit dentine during tusk formation. Cementum is present as a layer surrounding the proximal tusk and its main function is to attach the tusk root to the maxillary bone. Cementum is formed by cementoblasts, and is a softer material than dentine, with a higher water/collagen to mineral ratio (50:50, compared to dentine 5:95). Enamel, formed by ameloblasts, is the hardest tissue found in the mammalian body, and is almost entirely composed of mineral carbonated phosphate. The dentinal pulp is a mass of connective tissue containing nerves and blood vessels, as well as the ondontoblasts.

Ivory derives from the tusks (upper incisors) of animals from the Order Proboscidea. Tusk structures of elephants and mammoth are broadly similar. However, at a microscopic level, there are differences in the density of dentinal tubules [6, 8, 11, 12]. The dentinal tubules in mammoth ivory are more closely packed together than those in modern elephants [11], perhaps relating to behavioural differences which subjected the mammoth tusks to higher loading, such as more fighting or lifting. The Schreger pattern is also different in elephant and mammoth ivory.

The taxonomic Order Proboscidea encompasses mammals with trunks and tusks, such as the extant African bush elephant (*Loxodonta africana*), African forest elephant (*Loxodonta cyclotis*) and the Asian elephant (*Elephas maximus*), as well as many extinct species including the woolly mammoth (*Mammuthus primigenius*) [13]. Elephants and mammoths initially grow deciduous tusks that reach just 5 cm in length. These tusks fall out after one year and the permanent tusks continue to grow throughout the elephant’s lifetime. Initially, the dentine of a permanent tusk is covered with a thin peripheral layer of cementum and enamel; over time these layers of cementum and enamel wear off through use [14]. Both male and female African elephants and mammoths have tusks, while female Asian elephants lack tusks (or have small ‘tushes’). There are sex differences in the tusks of African elephants; male tusks are larger and increase in circumference and length throughout life, whereas female tusks are smaller and do not increase in circumference after maturity. In addition, after puberty, the pulp cavity of females begins to fill in with cementum, whereas in males it increases in size with age [15]. Similar differences have been noted in mammoths [16]. Tusks in both sexes are used in feeding and competitively for dominance. Elephants are known to have a dominant tusk side; the tusk on the dominant side is often shorter than the non-dominant tusks as it is worn down through greater use [17].

Ivory products have historically been popular worldwide. Research conducted by the Worldwide Wildlife Fund has studied consumer behaviour to identify reasons, culturally and practically, why the ivory trade still exists [18–20]. The study showed that buyers are most likely to be women with medium to high incomes that live in smaller Chinese cities [20]. These individuals purchase ivory for a number of reasons, mostly stemming from the historical precedent of ivory as a status symbol in the far East, due to its rarity and value, and that it is seen as a safe investment, similar to that of purchasing artwork. Ivory is also commonly used in traditional medicines, in jewellery, ornaments, small carvings and figurines, and was historically used in the production of objects such as piano keys and billiard balls. While the use of elephant ivory has decreased due to increased protection and conservation, exploitation of the Siberian permafrost and the organised efforts of the ‘mammoth hunters’ have allowed the mammoth ivory market to flourish.

In 2017, Palkopolou *et al*. [13] mapped the genomes data of extinct and living elephants. Although it has been estimated that there have been at least 200 different species of Proboscidea, of which about 40 were elephants, the only living members of this family now are two species of African elephant, *Loxodonta cyclotis* and *Loxodonta africana*, and one species of Asian elephant, *Elephas maximus* (Table 1) [13, 21]. The 2016 African Elephant Database survey estimated a total of 410,000 elephants remaining in Africa, a decrease of approximately 90,000 elephants from the previous 2013 report [22]. Although the percentage decline in Asian elephants as a result of illegal poaching is lower, as females do not have tusks, there has been a 50% decline over the last three generations of Asian elephants.

**Table 1:** A partial list of extinct and extant elephant species. The list of mammoths details the estimated date of extinction, and the list of modern day elephants gives details of remaining population size and International Union for Conservation (IUCN) of Nature Red List Assessment.

**Extinct Species**	**Date of Extinction**
*Mammuthus primigenius*	4000 BP
*Mammuthus columbi*	10,900 BP
*Paleoloxodonta antiquus*	30,000 BP
*Mammuthus americanum*	10,000 BP
*Mammuthus trogontherii*	200,000 BP
**Extant species**	**Population size, IUCN status**
*Elephas maximus*	50,000, endangered
*Loxodonta cyclotis*	<30,000, critically endangered
*Loxodonta africana*	550,000, endangered

The United Nations Office on Drugs and Crime recommends a variety of laboratory techniques to be used in assessing the legality of ivory in trade [23]. These include Schreger line analysis, Fourier transform infrared (FTIR) spectroscopy, DNA and mtDNA analysis, isotope analysis and Raman spectroscopy, though the recommendations heavily focus on the use of DNA/mtDNA and isotope analysis [23].

The primary non-destructive method for differentiating between elephant and mammoth ivory is based on the difference in the Schreger pattern [23]. Schreger lines can be divided into outer and inner, depending on whether they are found towards the surface or the medulla of the tusk. Outer Schreger lines can be used to distinguish between elephant and mammoth ivory due to a difference in their angles. In elephant ivory, the Schreger lines have an average angle of intersection of 115° and form a characteristic "V" shape. In mammoth ivory, the Schreger lines are more angled and form a "U" shape with intersections of less than 90°. However, the inner Schreger lines are not useful in the identification of ivory species. A database of the convex and concave Schreger lines was published identifying the differences between elephant and mammoth ivory [4]. However, this method of identification requires a perfect transverse section of tusk, perpendicular to the axis of the tusk [24]. In addition, ivory from another extinct proboscidean, the mastodon (*Mammut spp*.), presents obtuse Schreger angles similar to that of elephant [23]. This creates the possibility that well-preserved mastodon ivory could be mistaken as elephant ivory. Ultimately, Schreger line analysis can only differentiate between elephant and mammoth ivory, and other methods are required for finer-level identification of elephant species and subspecies. In cases where orientation of a tusk sample is difficult, DNA identification or radiocarbon dating may be sought–but these studies are costly, time consuming and destructive.

FTIR and Raman spectroscopy both work through the detection of vibration in molecules, based on either the infra-red absorption or Raman scattering [25]. Up until ten years ago, Raman spectroscopy was less widely used than FTIR due to issues with sample degradation and fluorescence, although modern technology has resolved many of these issues [26]. Modern Raman spectrometers are relatively simple to use and are non-destructive to biological specimens [27]. The use of this technique is well documented in the analysis of bone health [28–30], and spatially offset Raman spectroscopy has been used to give detailed information as to the biochemistry of a specimen below the surface area of a tissue [31]. This technique is non-destructive, and can be used *in-vitro*, *in-vivo* or *ex-vivo* [31].

Raman spectroscopy is a method of measuring and quantifying changes in energy of a light (using a laser) as it is scattered from a material [31, 32]. As the light (photons) interact with molecular bonds, providing energy for them to vibrate, energy is lost or gained, which results in a shift in wavelength. The output data, containing information about the different changes in light, is referred to as a Raman ‘fingerprint’ or ‘a spectrum’ [31]. Biochemical components, such as the organic and mineral components of calcified tissue samples can be identified by interpretation and analysis of the peaks present [28, 30, 32]; further multivariate analysis, such as Principle Component Analysis (PCA) and Linear Discriminant Analysis (LDA) can be used to elucidate changes across the spectral range [32–35].

For example, a higher collagen to phosphate ratio allows more elasticity of the sample. *L*. *cyclotis* ivory has a complex internal structure with a pronounced "criss-cross" pattern of collagen fibres, which gives the tusk greater strength and durability [36], and this ‘hardness’ is preferred in the Japanese ivory market [37]. There is little preference in China, where the ‘softer’ ivory of both *L*. *africana* and *E*. *maximus* is used. Ivory from these species is more brittle and liable to crumble, possibly due to a lower collagen to phosphate ivory ratio compared with *L*. *cyclotis* [37]. There are also observable differences in the colour of ivory from Loxodonta species. The savannah elephant has cream-coloured ivory, whereas the tusks from the forest elephant are described as having a pink tinge [38]. Such ‘pink ivory’ is highly valued in Japan [39], where it is often used for name seals, known locally as ‘hanko’ or ‘inkan’. Raman spectroscopy has previously been used to analyse ivory specimens, in order to assess the biodeterioration of samples [40], the age of elephants [41], and to distinguish between real and fake ivories [42]. Spatially offset Raman spectroscopy has been utilised to identify ivory concealed below a coating intended to avoid detection [42]. As the technique is non-destructive and does not require any sample preparation there is potential for it to be applied in the identification of mammoth and elephant ivory at customs worldwide to aid in the enforcement of ivory trade bans [37, 43–45].

In this paper, it is hypothesised that mammoth and elephant ivory can be distinguished using Raman spectroscopy because of the differences in their biochemical composition. This hypothesis will be tested by measuring the differences in mineralisation and collagen composition of tusks.

## Methods

### Specimens

Samples of mammoth and elephant tusks were kindly loaned by the Natural History Museum, London, UK (Table 2).

**Table 2:** Sample list. Samples were loaned by NHM, London. In total, three modern elephant samples and eight mammoth samples were analysed.

	NHM Specimen Number	Species	Description
***Extant Elephant samples***			
1	BMNH GERM 707.e	*Elephas maximus*	Section of ivory tusk
2	BMNH ZD 2002.40a-d	*Loxodonta spp*.	4 Bangles donated by Customs and Excise, 2003
3	BMNH 2002.39	*Loxodonta spp*.	4 Bangles donated by Customs and Excise, 2003
***Extinct Elephant samples***			
1	PV M 104580	*Mammuthus primigenius*	Fragment of tusk with one sawn end. The other end is fragmented
2	PV M 96540	*Mammuthus primigenius*	Tusk fragment with two sawn off ends. The ivory trader’s mark is present on the side
3	PV M 1620	*Mammuthus primigenius*	Transverse section across the portion of the tusk containing the pulp cavity
4	PV M 10968	*Mammuthus primigenius*	Fragment of tusk cut open to reveal the internal structure. Outer surface is rough with some pale green patches
5	PV M 10968	*Mammuthus primigenius*	Fragment of tusk cut open to reveal the internal structure. Outer surface is rough with some pale green patches. A portion of the ivory trader’s mark can be seen
6	PV M 104581	*Mammuthus primigenius*	Curved fragment of tusk with smooth, sawn ends. Surface is rough and has a very weathered appearance. A very small portion of the ivory trader’s mark can be seen
7	PV M 104579	*Mammuthus primigenius*	Distal end of tusk with one sawn end. A portion of the ivory trader’s mark can be seen along its edge
8	PV OR 44917	*Mammuthus primigenius*	Transverse section of one half of an incisor

**Fig 1:**
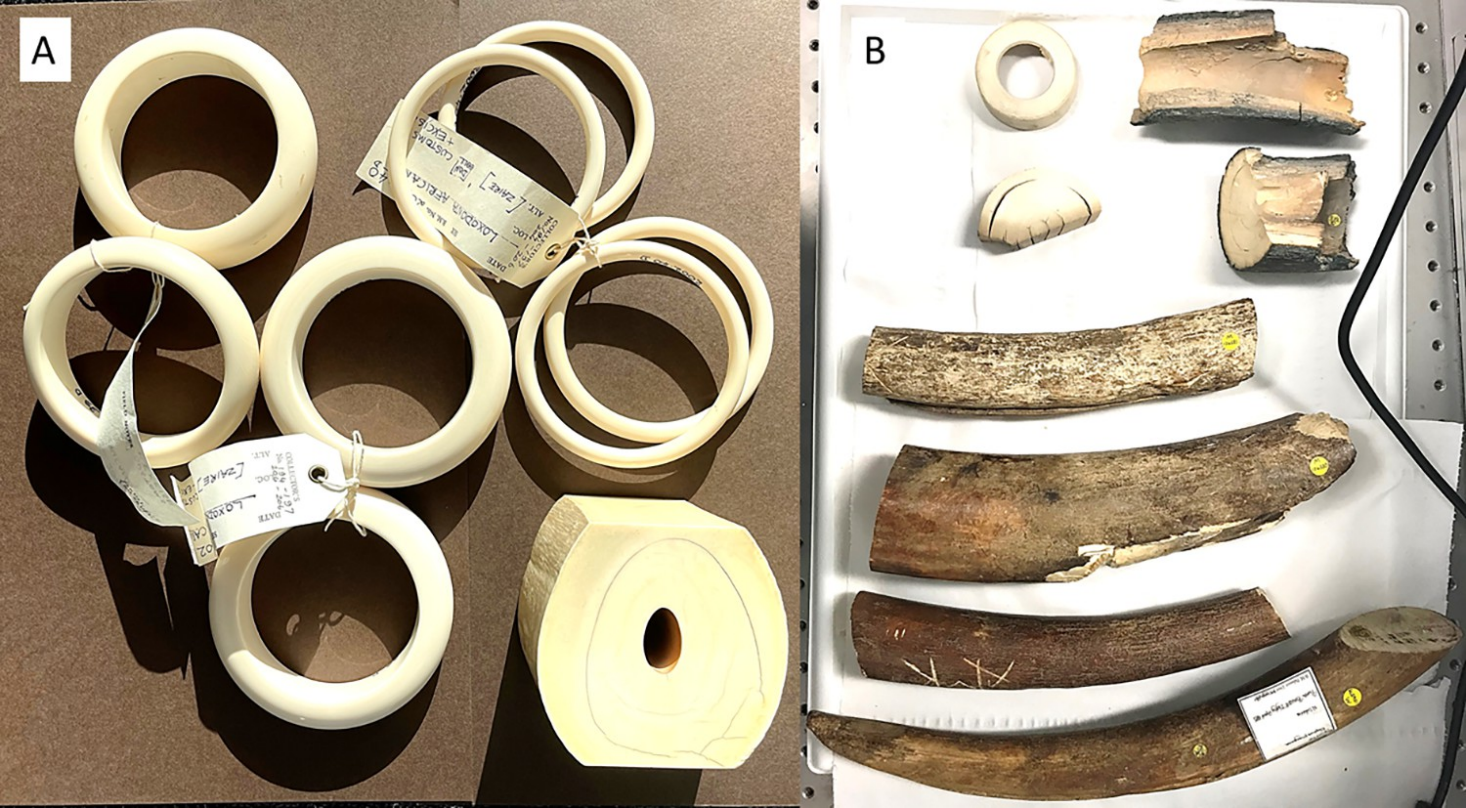
Ivory samples included in this project. **(A)** the *Loxodonta spp*. samples (8 bangles) and in the lower right corner is the single *Elephas maximus* sample and **(B)** the *Mammuthus primigenius* samples. A full description of each sample is given in Table 1.

All of the mammoth tusks (Fig 1B) were from the species *Mammuthus primigenius*, with samples originating from either the Lyakhov Islands and near the Yenisei river, Krasnoyarsk (Siberia, Russia), and are of Late Pleistocene age. The elephant ivory samples (Fig 1A) consisted of a cross section of an *Elephas maximus* tusk and eight carved ivory bangles from *Loxodonta spp*. The samples had been identified by staff at the Natural History Museum based on geographical origin and gross appearance. To the best of our knowledge none of the samples had been treated or coated.

### Raman spectroscopy

Spectra were acquired from each ivory sample with an inVia Raman micro spectrometer (Renishaw Ltd, Gloucestershire, UK) equipped with an Olympus 50×/0.5 long working distance objective lens and a 785nm laser, 200mW at source with 1200 l/mm grating. The laser power at the sample was ~10 mW. Each spectrum was collected for 60s (6 × 10 s accumulations) in the spectral range ~600–1700 cm^-1^. Independent spectra were collected at a minimum of ten different locations, more than 10 μm apart, for each sample to provide replicates and to account for any heterogeneity. Further spectra were acquired using line maps from the medulla to the cortex of a tusk on one mammoth sample. The spectra were not collected from areas of tusks that had visible debris or damage. In total, 272 spectra were obtained from 8 *Mammuthus primigenius* samples, 203 spectra from *Loxodonta spp*. and 17 spectra were obtained from *Elephas maximus*.

### Data analysis

Data was analysed with Matlab (v2021a, The Mathworks, Inc., Natick, MA, USA). All spectra were baseline corrected using a sixth order polynomial subtraction and vector normalised using a previously published script [33, 46]. Principal component analysis (PCA) was performed on this data, and up to 10 principal components were generated for each analysis. Data were classed by species and/or genus for the purposes of the analysis.

Univariate analysis of the phosphate (960 cm^-1^) to amide I (1660 cm^-1^) peak, and the phosphate (960 cm^-1^) to amide III (1240–1260 cm^-1^) peak intensity ratios were performed using Microsoft Excel (Microsoft Corporation) to compare the mineral to collagen ratio of samples from elephant and mammoth tusks. These peaks were chosen as significant peaks consistently present in spectra from every sample. In addition, peak intensity ratios of carbonate (1060 cm^-1^) to phosphate (960 cm^-1^) were used to look at the carbonate substitution. A one-way ANOVA for each peak intensity ratio was performed using GraphPad Prism (v10.0.0 for Windows, GraphPad Software, Boston, Massachusetts USA). The full width at half maximum (FWHM) of the phosphate peak (960 cm^-1^) was calculated using SpectraGryph (v1.2.16.1, 2023) to compare crystal maturity.

When analysing the data from the line map, the spectra were grouped in 14 classes (each class representing approximately 0.5 cm of travel) and PCA-Linear discriminant analysis (PCA-LDA) was performed.

## Results

**Fig 2:**
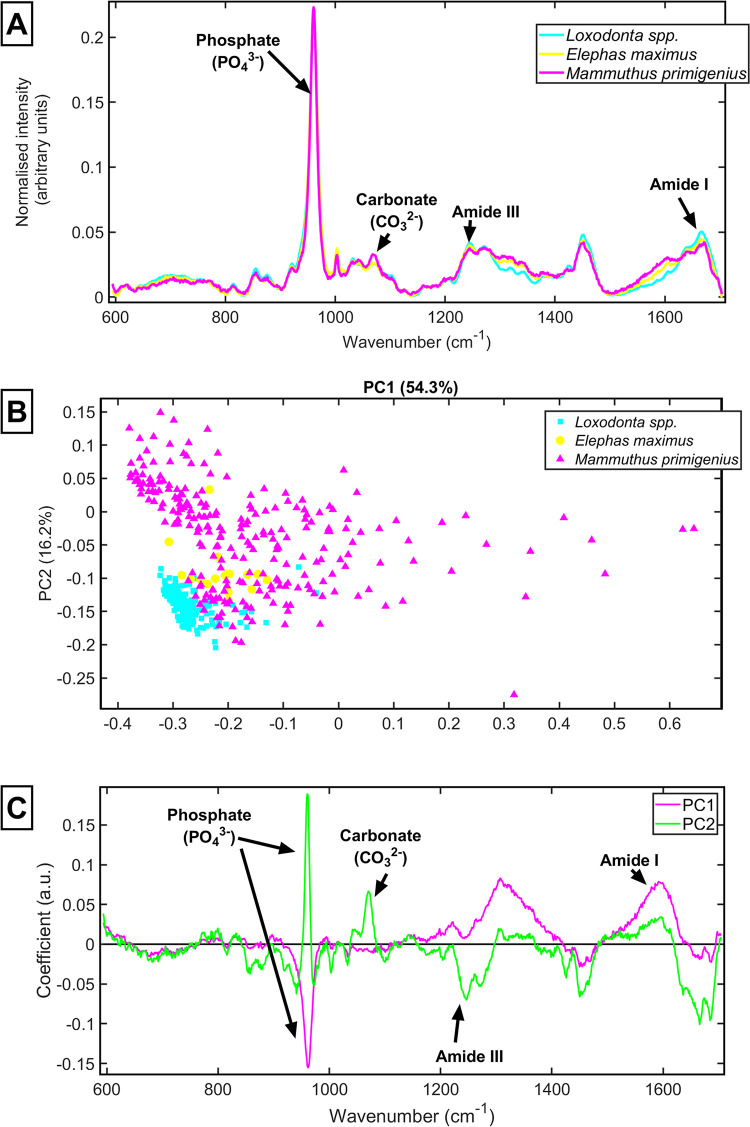
Average Raman spectra from the ivory sample of *Mammuthus primigenus*, *Loxodonta* and *Elephas maximus*. [**A**] Average spectrum from each tusk sample, Mammuthus primigenus (magenta), *Loxodonta spp*. (cyan) and *Elephas maximus* (yellow); [**B**] PCA scores plot of *Mammuthus primigenus* (magenta triangles), *Loxodonta spp*. (cyan squares) and *Elephas maximus* (yellow circles); [**C**] Loadings plot showing differences in PC1 and PC2.

The average spectra of each sample show that the biochemical composition of the samples is broadly similar (Fig 2A). The quality of the spectra from the mammoth ivory, demonstrate that the mammoth ivory is well preserved, as there are prominent organic collagen peaks.

The results reveal similarities and differences in the spectral ‘fingerprint’ of ivory from different species of mammal. PCA scores plots (Fig 2B) reveal distinct groupings of different species with some inter-sample variation.

Fig 2A presents the average spectra from each of the 11 samples analysed in this study, categorised by species. All samples demonstrate a well-preserved organic component with prominent amide peaks. The PCA scores plot (Fig 2B) demonstrated some separation between the species, and some within-species variation; the largest distinction is between the chemistry of ivory from *Loxodonta* and *Mammuthus primigenius*. Fig 2C, a PCA loadings plot, suggests the largest contribution to the differences are at the phosphate (960 cm^-1^), amide III (1240–1260 cm^-1^) and amide I (1650 cm^-1^) peaks.

**Fig 3:**
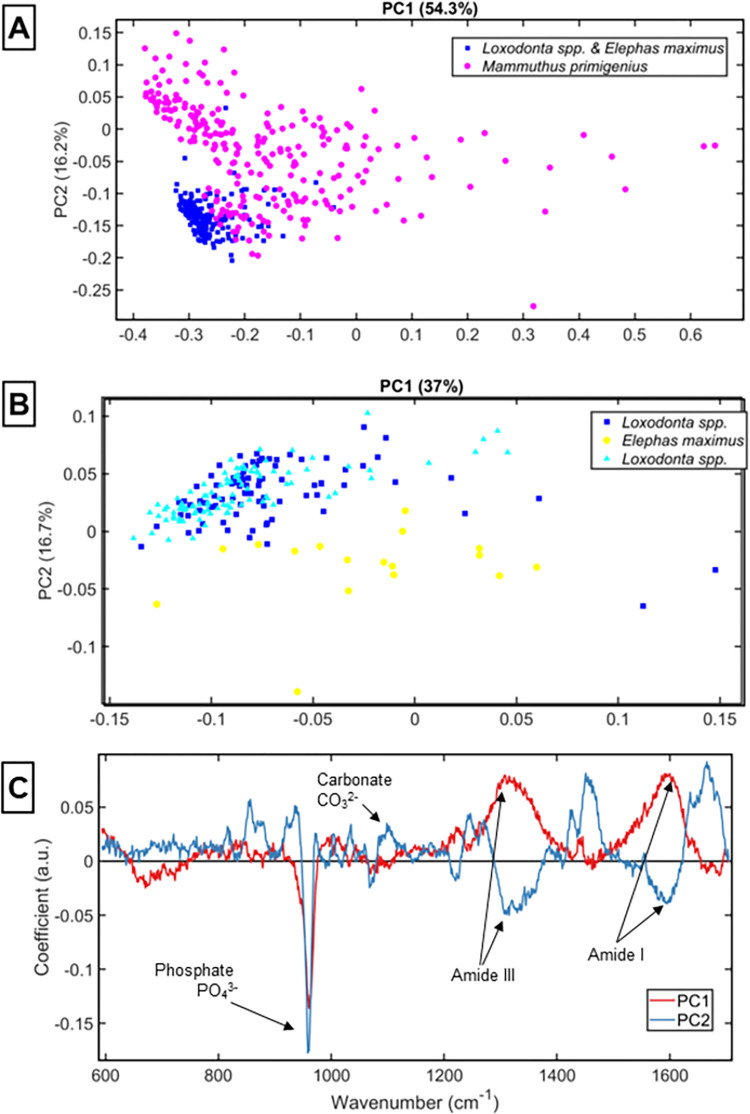
[**A**] PCA scores plot of the ivory samples *Mammuthus primigenius* [magenta] and living elephants [blue] and [**B**] PCA plot of from each tusk sample *Loxodonta spp*. (cyan and blue) and *Elephas maximus* (yellow); [**C**] PCA Loadings plot corresponding to [B].

The data demonstrates some distinction between the mammoth and elephant ivory, with the corresponding PCA loadings plot showing differences in PC1 at the phosphate (961 cm^-1^), lipid (1300 cm^-1^) and left hand side of amide I (1590 cm^-1^) regions, and PC2 contains notable contributions from the phosphate (960 cm-1), middle of the amide I peak (1665 cm^-1^), amide III (1250 and 1270 cm^-1^), carbonate (1070 cm^-1^) and CH2 (1250 cm^-1^) peaks (Fig 3C). This means that the strongest variation between all three species, identified from differences along PC1, is due to specific organic contributions and the wavenumber immediately to the right of the centre of the hydroxyapatite peak. *Mammuthus primigenus* is further separated along PC2 due to contributions from the centre of the phosphate peak, carbonate peak and several collagen peaks (Fig 3C).

**Fig 4:**
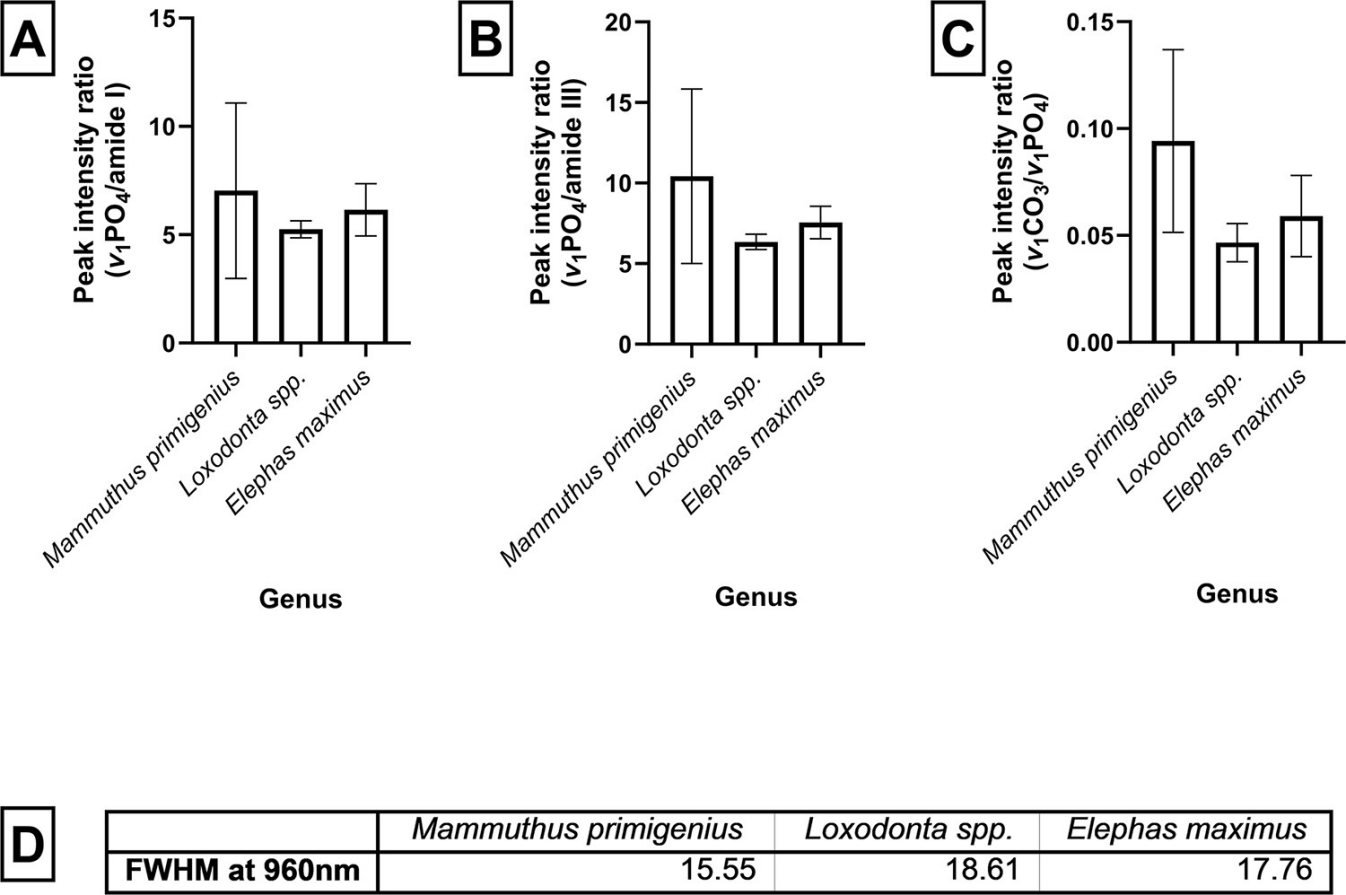
Univariate analysis of **A)** peak intensity ratios of phosphate (960 cm^-1^) to amide I (1660 cm^-1^) from ivory samples taken from *Mammuthus primigenius*, *Loxodonta spp*. and *Elephas maximus*. The error bars show 1 standard deviation from the mean. An ordinary one-way ANOVA was performed F = 19.99, with a significant difference between the means (p<0.0001), R squared = 0.07559. **B)** peak intensity rations of phosphate (960 cm^-1^) to amide III (1240–1260 cm^-1^) from ivory sample taken from *Mammuthus primigenius*, *Loxodonta spp*. and *Elephas maximus*. The error bars show 1 standard deviation from the mean. An ordinary one-way ANOVA was performed F = 59.52, with a significant difference between the means (p<0.0001), R squared = 0.1958. **C)** peak intensity ratios of carbonate (1060 cm^-1^) to phosphate (960 cm^-1^) of ivory samples taken from *Mammuthus primigenius*, *Loxodonta spp*. and *Elephas maximus*. The bars show 1 standard deviation from the mean. An ordinary one-way ANOVA was performed F = 126.2, with a significant difference between the means (p<0.0001) R squared = 0.3404. **D)** The full width at half maximum (FWHM) of the phosphate peak (960 cm^-1^) was calculated to compare crystal maturity.

An analysis of the ratio between the phosphate and amide1 (Fig 4A) or amide III (Fig 4B) peaks provides the potential to differentiate between ivory between the species (Fig 4A–4C). The one-way ANOVA of the peak intensity ratios between amide I, amide III and carbonate peaks against the hydroxyapatite peaks (Fig 4A–4C) all demonstrated a statistically significant difference (p<0.0001) between the means of all three members of the Elephantidae family for each ratio calculated. Specifically, the *Loxodonta spp*. ivory samples possessed a higher ratio of collagen (amide I and amide III) to phosphate (Fig 4A and 4B).

**Fig 5:**
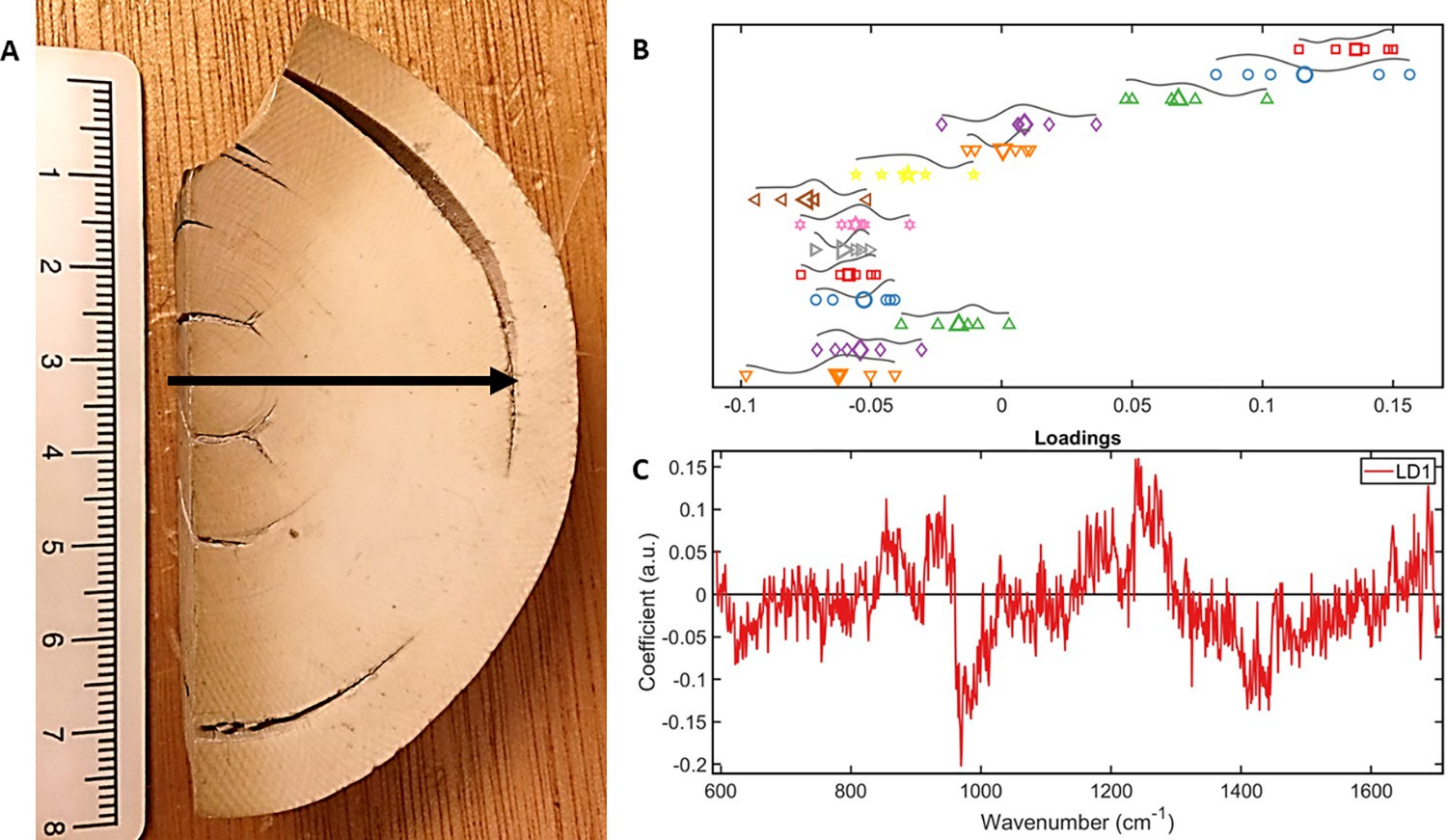
[**A**] A line map was created on a cross section of mammoth tusk. [**B**] The spectra were divided in 14 classes, each accounting for approximately 0.5 cm of the tusk radius. The 1D scatterplot demonstrates a consistent chemical composition of the tusk for the medulla up to the first 3.5 cm of the inner tusk, then changes in composition. [**C**] a loadings plot showing the biggest differences in spectra at the phosphate peak, suggesting that there is an increase in mineralisation towards the cortex.

The line map spectral analysis (Fig 5A–5C) performed on a cross section of mammoth tusk identifies differences in the hydroxyapatite peaks from cortex to medulla, suggesting an increase in mineralisation towards the cortex compared to the medulla.

## Discussion

The results of this study demonstrate that Raman spectroscopy possesses clear utility for the identification of ivory samples of unknown origin. This study has shown that differences in the average spectra between species can be identified using PCA, and that univariate analysis of the phosphate to amide I and amide III peaks can potentially be used to distinguish between species of the Elephantadiae genus, as can comparison of the carbonate to phosphate peaks. The higher mineral-to-collagen ratio of Elephas maximus could reflect the closer evolutionary divergence to Mammuthus species than of Loxodonta species. This study has also shown that Raman spectroscopy could also be used to identify whether an ivory sample is from the medulla or the cortex of a tusk. Furthermore, the analysis has highlighted the main spectral features that differ between and within species, largely related to the mineralisation profile. This could be further explored with larger sample sizes, particularly because differences in mineralisation of bone between species is well reported, and it is hypothesised that teeth could exhibit variation based on structure, function within a species, and the environment.

The inter-sample variation that was seen in the *Mammuthus* species could be due to age of the sample, age of the mammoth at time of death, differences in diet or climate conditions, or that it may have been subject to slightly different geological conditions which can have an impact on the tusk microstructure and therefore subsequent material properties, such that permafrost preserved ivory has a lower hardness than fresh material [47]. There is also evidence of post-mortem changes in bone samples after burial, whereby trace elements are exchanged between the bone and the surrounding material [49]. It is likely than this phenomenon also occurs in buried tusk samples.

In addition, in this study the *Loxodonta spp*. samples had been previously ground and polished; this means that there was an increased number of photons reflected and an improved signal-to-noise ratio. Many of the mammoth samples, however, had a rough surface and had not been polished, meaning they had a lower signal-to-noise ratio, which could partially explain the wider variation in the spectra obtained from the *Mammuthus primigenius* samples.

Preliminary work has suggested it is possible to tell the biological age of an individual elephant from which a tusk has been taken by comparing the collagen to bioapatite peak ratios of the samples [41]. Data taken from human research also suggests that Raman spectroscopy can be used in understanding the dating of mammalian calcified tissues [48–51]. However, to date, there has been limited research into the dating of tusks using Raman spectroscopy [52]. The authors hypothese that this could be possible to observe differences based on date via Raman spectra in one of two ways: either by the collagen to mineral degradation rate over time, or through the identification of proxy substances (a method used in dating historical art [53]) found either coating the ivory superficially, or that have been incorporated within the tusk matrix. The development of a non-destructive technique for dating ivory samples could aid in the differentiation of antique and newly created ivory artefacts and provide another powerful tool for the detection of illegal ivory across the globe.

A significant limitation of this study is that only a small number of ivory samples were analysed. This means there is a limited amount of information that is captured by the analysis. The work has yet to explore differences in the biochemistry between *Loxodonta africana* and *Loxodonta cyclotis*. *L*. *cyclotis* tusks are known to be straighter than those of *L*. *africana*, and their ivory is described as harder and pinkish in colour [54]. During the formation of dentine, there are many ions that can be substituted within the crystal structure, this can include anionic, such as F^−^, Cl^−^, SiO_4_^4−,^ and CO_3_^2−,^ or cationic substitutions such as Na^+^, Mg^2+^, Fe^2+^, K^+^, Sr^2+^, Zn^2+^, Ba^2+^, Al^3+^ [10]. It is possible that such substitutions could be responsible for the ‘pinkish’ tinge to the tusks of *L*. *cyclotis*, and that this different in biochemical composition could be used to separate *L*. *africana* and *L*. *cyclotis* spectroscopically. Future work should aim to further understand the differences in chemical structure of tusks between these extant elephants, and could also benefit from genetic analysis of tusks alongside spectroscopic analysis, to ensure accurate species identification.

It is possible that some of the ‘interspecific’ differences identified here, particularly between the eight mammoth samples, could be partly or even largely due to differences in the sex or age, or taphonomic changes to collagen over time. Further research with larger sample sizes is needed to assess levels of variation, both within and between species, and the use of photochemical bleaching prior to spectral acquisition could be used to reduce background fluorescence. However, despite the limitations imposed by sample size, the major pattern in the analysis of our data appears to reflect interspecific differences.

This study used PCA and PCA-LDA as multivariate analytical techniques [33], though other methods, such as multiple linear regression, cluster analysis and partial least squares regression, are used in the analysis of Raman spectroscopic data sets [55]. A future comparison of these methods may prove useful in ensuring correct species classification.

In conclusion, Raman spectroscopy is a promising tool for the identification of ivory. While this study utilised a large, laboratory-based inVia Raman spectrometer, a recent study has demonstrated that smaller, more portable, mobile Raman spectrometers could offer a similar quality of data [44]. Handheld Raman spectrometers have been used for several years in the study of bone tissue [31, 56] and are regularly used in industry for the purposes of raw material verification and unknown substance identification [57].

Further work is needed to assess intra- and interspecific variation and to compile a functional database of reference spectra that could be used for identifying unknown ivory samples. An average spectral signature of each species could be added to the CITES trade database [58] or the UNDOC guidelines for ivory identification [59] for rapid consultation at customs points around the globe. This could form a quick and easy method of determining ivory species to help combat illegal trade. Increased surveillance and monitoring of samples passing through customs worldwide using Raman spectroscopy could act as a deterrent to those poaching endangered and critically endangered species of elephant.
